# The National Cancer Aid monitoring (NCAM-online) of ultraviolet radiation risk and protection behavior: a population-based observational trend study with four annual online survey waves

**DOI:** 10.1186/s12889-024-19938-0

**Published:** 2024-09-08

**Authors:** Katharina Diehl, Eckhard W. Breitbart, Yvonne de Buhr, Tatiana Görig

**Affiliations:** 1 Professorship of Epidemiology and Public Health, Department of Medical Informatics, Biometry and Epidemiology, Friedrich-Alexander-Universität Erlangen-Nürnberg (FAU), Waldstraße 6, 91054 Erlangen, Germany; 2 Bavarian Cancer Research Center (BZKF), Erlangen, Germany; 3 Comprehensive Cancer Center Erlangen-European Metropolitan Area of Nürnberg (CCC ER-EMN), Erlangen, Germany; 4 Arbeitsgemeinschaft Dermatologische Prävention, Buxtehude, Germany

**Keywords:** Ultraviolet radiation, Tanning behavior, Indoor tanning, Tanning bed, Sunbed, Sun protection, General population, Germany

## Abstract

**Background:**

Ultraviolet (UV) radiation is the most important risk factor for skin cancer development. Sunlight is the main source of UV radiation in the general population. In addition, tanning beds are a source of artificial UV radiation. Since the incidence of skin cancer is increasing worldwide, it is necessary to monitor UV-related risk behaviors such as intentional indoor and outdoor tanning, as well as sun protection behavior in the general population and specific subgroups and settings. This is the aim of the National Cancer Aid Monitoring online (NCAM-online), a continuation and further development of the NCAM.

**Methods:**

The NCAM-online is a longitudinal trend study consisting of four annual survey waves. Each year, 4,000 individuals aged 16–65 years living in Germany will be surveyed using online questionnaires. Each year, intentional indoor and outdoor tanning will be assessed. In addition, varying specific topics regarding skin cancer prevention, such as the utilization of skin cancer screening, will be addressed in the questionnaires.

**Discussion:**

The findings of the NCAM-online will provide an important basis for the German Cancer Aid and Working Group on Dermatologic Prevention (Arbeitsgemeinschaft Dermatologische Prävention, ADP) to develop targeted prevention campaigns and projects aimed at preventing skin cancer. The explorative nature of the NCAM-online allows for the identification of new potential starting points for prevention and education. In addition, the longitudinal design allows for a description of the trend in the prevalence of intentional tanning. For tanning bed use, representative trend data from 2012 are available for Germany, to which NCAM-online will add annual data until 2027.

## Background

The incidence of skin cancer is increasing worldwide, and it is the most common type of cancer in the white population [1]. The core topic of the National Cancer Aid Monitoring online (NCAM-online) is intentional indoor and outdoor tanning. Both aspects are risk factors for the development of skin cancer, including melanoma and nonmelanoma skin cancer, primarily squamous cell carcinoma and basal cell carcinoma [2–4]. The International Agency for Research on Cancer (IARC) has classified both solar radiation and tanning beds as carcinogenic to humans [5]. By that time, the WHO had been warning against the use of tanning beds for several years [6]. Given the increasing incidence of skin cancer in Germany [7], it is important to monitor risk factors for skin cancer.

Although indoor and outdoor tanning are associated with health risks that are not limited to the potential development of skin cancer, many people in Western countries expose themselves intentionally to the sun (e.g., for Europe [8, 9]) or use tanning beds (e.g., for Europe [10, 11]). One of the most important reasons is the perceived beauty ideal: tanned skin is seen as attractive and healthy [12, 13]. To compare data on tanning between countries, develop targeted education campaigns, and monitor the success of prevention, the collection of population-based survey data is necessary. In particular, trend data are important for understanding the development of intentional tanning and its determinants over time.

## Development of the NCAM-online

NCAM-online represents both a continuation and a modernization of previous studies by the study group and others. Following smaller regional [14–16] and national surveys [17], the SUN Study 2012 (Sunbed Use: Needs for Action Study 2012) was the first to provide nationally representative data on sunbed use in Germany [18–23]. Based on the SUN Study 2012, the National Cancer Aid Monitoring (NCAM) was developed and established in 2015. The NCAM is a longitudinal monitoring of ultraviolet (UV)-related risk behavior in the German population, similar to those that have existed for other oncological risk factors such as tobacco and alcohol consumption since the 1970s.

With the help of the NCAM, it was possible to observe the use of tanning beds over time in Germany [24–26]. In eight annual representative survey waves, 3,000 people aged 14 to 45 years [NCAM I; waves 1 to 4] and 4,000 people aged 16 to 65 years [NCAM II; waves 5 to 8] were surveyed by telephone [26]. The NCAM I and NCAM II provided the first trend data for UV-related risk behavior in the German population. The study is unique worldwide.

In addition to the prevalence of intentional indoor and outdoor tanning, the NCAM can be used to describe and monitor the individual characteristics of tanners and risk perceptions over time [24, 26–28]. Moreover, data on sun protection behavior in the German population [29, 30] and in subgroups such as outdoor workers [31–34], parents and children [35, 36], and outdoor athletes [37] have been collected. Psychological aspects, such as tanning addiction [38], attractiveness-related motives for tanning behavior [39], and the perceived risk of developing skin cancer [40], were also explored using NCAM data. The NCAM forms an important basis for the interventional work of the German Cancer Aid Foundation and other institutions involved in skin cancer prevention.

The NCAM-online represents a further development of the study group’s previous studies and will be based on representative online surveys rather than telephone surveys. This change in the method of data collection can be attributed to several reasons. First, online surveys are more cost-effective and resource-efficient than telephone surveys, and they are independent of time [41, 42]. Second, trust in telephone surveys has declined in recent years due to increased marketing and fraud calls [43], resulting in low response rates in many studies. Third, online surveys offer the opportunity to use more complex survey instruments and, for example, to integrate images or multimedia, which is an advantage over telephone surveys. Fourth, interviewer effects can be excluded in online surveys [44]. Fifth, there is an increase in households without a landline telephone, especially among young people [45].

## Aim of the study

**Fig. 1:**
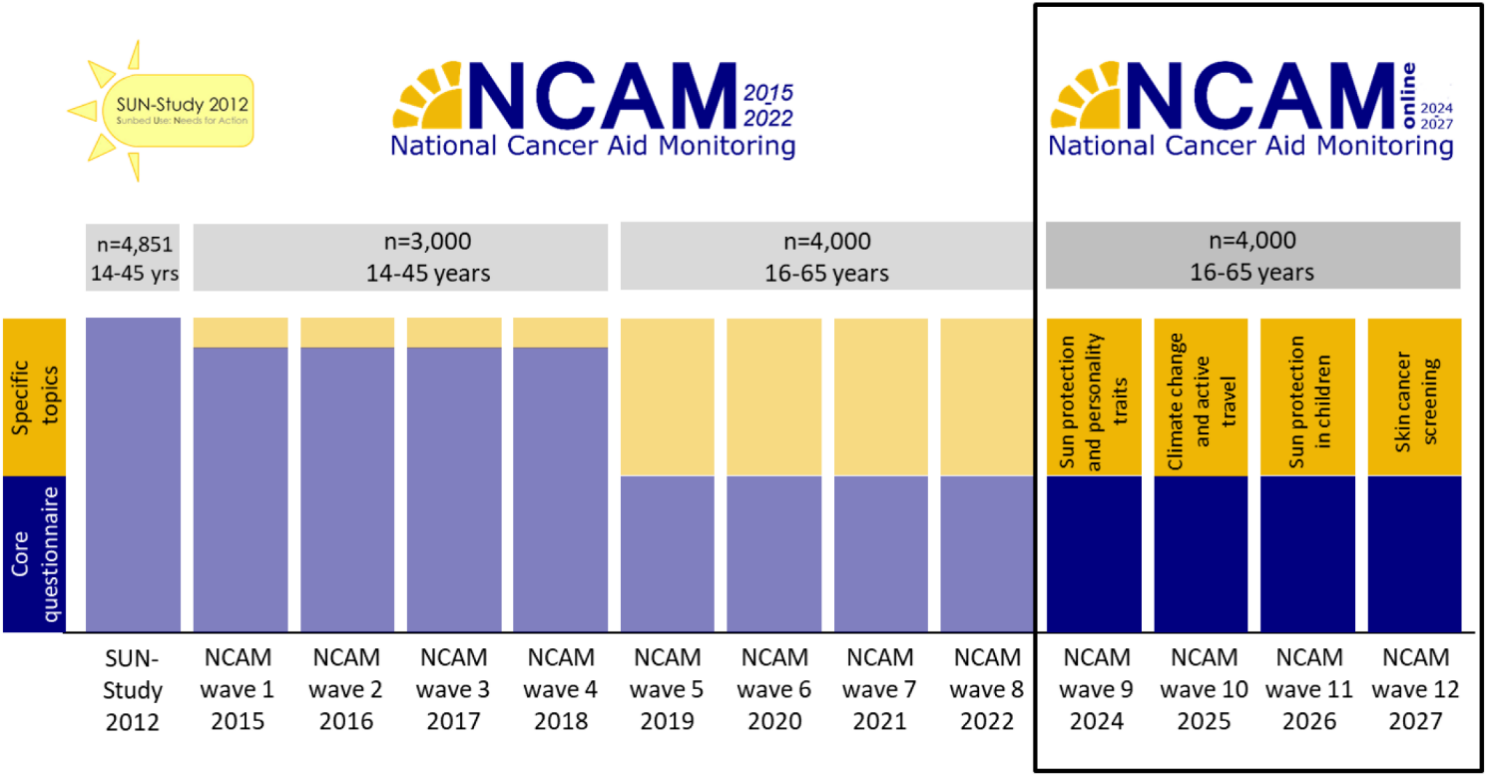
Overview of the NCAM-online and its connection to previous representative studies in Germany. *Legend* NCAM = National Cancer Aid Monitoring; SUN = Sunbed Use: Needs for Action

The aim of the NCAM-online is to monitor UV-related risk and protection behaviors in the German population aged 16–65 years. In four annual waves (wave 9 in 2024, wave 10 in 2025, wave 11 in 2026, and wave 12 in 2027), indoor and outdoor tanning will be assessed for 4,000 individuals in each wave to monitor trends in intentional tanning. In addition, each wave will focus on specific topics regarding skin cancer prevention, as described below (Fig. 1).

### Wave 9

The focus during this survey wave will be on sun protection to gather current data in the German population that can be compared to previous waves of the NCAM (waves 1 and 5). In addition, the association between sun protection behavior and personality will be studied. Previous studies have revealed associations between health and health-related risk behaviors on the one hand and personality on the other. For instance, extraverted individuals were more likely to consume tobacco products and alcohol [46]. In contrast, conscientiousness was associated with healthy behavior, e.g., less consumption of tobacco, drugs, and alcohol, as well as a healthy diet and physical activity [47]. However, the associations between personality structures and tanning and sun protection behaviors have been neglected thus far. A study conducted in the 1990s among female students showed that conscientious students were more likely to use sun protection measures [48]. A more recent study revealed that neuroticism and extraversion were associated with tanning addiction [49]. In our study, we hypothesize that (1) extraversion is positively associated with UV-related risk behavior (both the use of tanning beds and intentional tanning in the sun) and negatively associated with sun protection behavior, and (2) conscientiousness is positively associated with sun protection behavior.

### Wave 10

This wave focuses on two separate aspects. First, we will assess the general population’s knowledge about the association between climate change and UV-related risk and protection behavior. Climate change has a direct impact on UV-related health damage, particularly skin cancer; therefore, adapted sun protection behaviors are needed. Climate change can increase population UV exposure in different ways. For example, low ozone events can occur more frequently and can lead to unexpectedly high levels of UV radiation in spring [50, 51] when the skin is still particularly UV sensitive [52]. In addition, in recent decades, the UV index has increased significantly during the spring and summer months [51, 53], the duration of sunshine throughout the year has increased [54], and the daily total UV irradiance that causes sunburn has increased [55]. With regard to this aspect, we hypothesize that (1) knowledge about the association between climate change and UV-related health damage is limited, (2) knowledge about the possible effects of climate change on individual sun protection behavior is inadequate, and (3) knowledge and awareness of climate change-related adaptation to UV exposure and protection behavior differ according to sociodemographic characteristics.

Second, the questionnaire will focus on individual’s sun protection behavior during active travel in daily life [56]. Active travel describes patterns of commuting by walking or cycling, for instance, to work or leisure activities, as part of daily routine [57]. In NCAM wave 6, sun protection behavior during outdoor sporting activities was already examined and found to be inadequate [37]. However, the role of sun protection during active travel remains unclear. It will also be explored whether active travel is avoided and replaced by using a car or whether additional sun protection measures are taken (e.g., wearing appropriate clothing). With regard to this aspect, we hypothesize that (1) sun protection behavior during active travel is inadequate, (2) sun protection during active travel differs by sex, (3) those who engage in active travel with their children are more likely to protect their children from the sun than themselves, and (4) those who engage in active travel with their children are more likely to protect themselves than people who actively travel without children.

### Wave 11

The questionnaire will focus on sun protection behavior in children from the parents’ perspective and will connect to an earlier wave of the NCAM [36]. In wave 6, we explored sun protection behavior in children and its association with parental sun protection and role modeling [35, 36]. Based on these findings, sun protection behavior will be studied in more detail. In addition, the risk of sunburn in children will be investigated in specific situations in daily life [58]. Moreover, the role of the UV index in sun protection behavior will be explored. Our hypotheses in this regard were as follows: (1) in intentional situations of sun exposure, greater precautions are taken with regard to sun protection of children than in unintentional situations, and (2) sunburn in children is more likely to occur in unintentional situations of sun exposure than in intentional situations.

### Wave 12

This wave focuses on skin cancer screening. Standardized skin cancer screening was introduced in Germany in 2008. Statutory health insurance covers the costs of biennial screening for members aged 35 years and above. Skin cancer screening can be performed by dermatologists and general practitioners. The NCAM wave 5 revealed deficits in the screening from the patients’ perspective [59]. Therefore, our aim was to assess the perception and use of skin cancer screening to compare trend data. This can give an impression of whether the engagement of relevant expert associations in recent years has contributed to improvements and more standardized examination and advisory practice. The focus of the questionnaire will also be on self-examination of the skin. We hypothesize that (1) certain body areas (e.g., the scalp and genital area) are examined less frequently during whole-body examination than other areas, (2) dermatologists examine body areas recommended to be examined during screening more frequently than general practitioners, (3) the offer of advice on the prevention of skin cancer by physicians performing skin cancer screening is inadequate, and (4) dermatologists offer alternative skin cancer examinations instead of statutory skin cancer screening and bill these as a self-paying service.

## Implications

The NCAM-online may have a strong impact on skin cancer prevention. The findings will be relevant for the prevention work of the German Cancer Aid and the Working Group on Dermatologic Prevention (Arbeitsgemeinschaft Dermatologische Prävention, ADP). Based on these findings, targeted prevention campaigns and projects aimed at preventing skin cancer will be developed or refined.

**Fig. 2:**
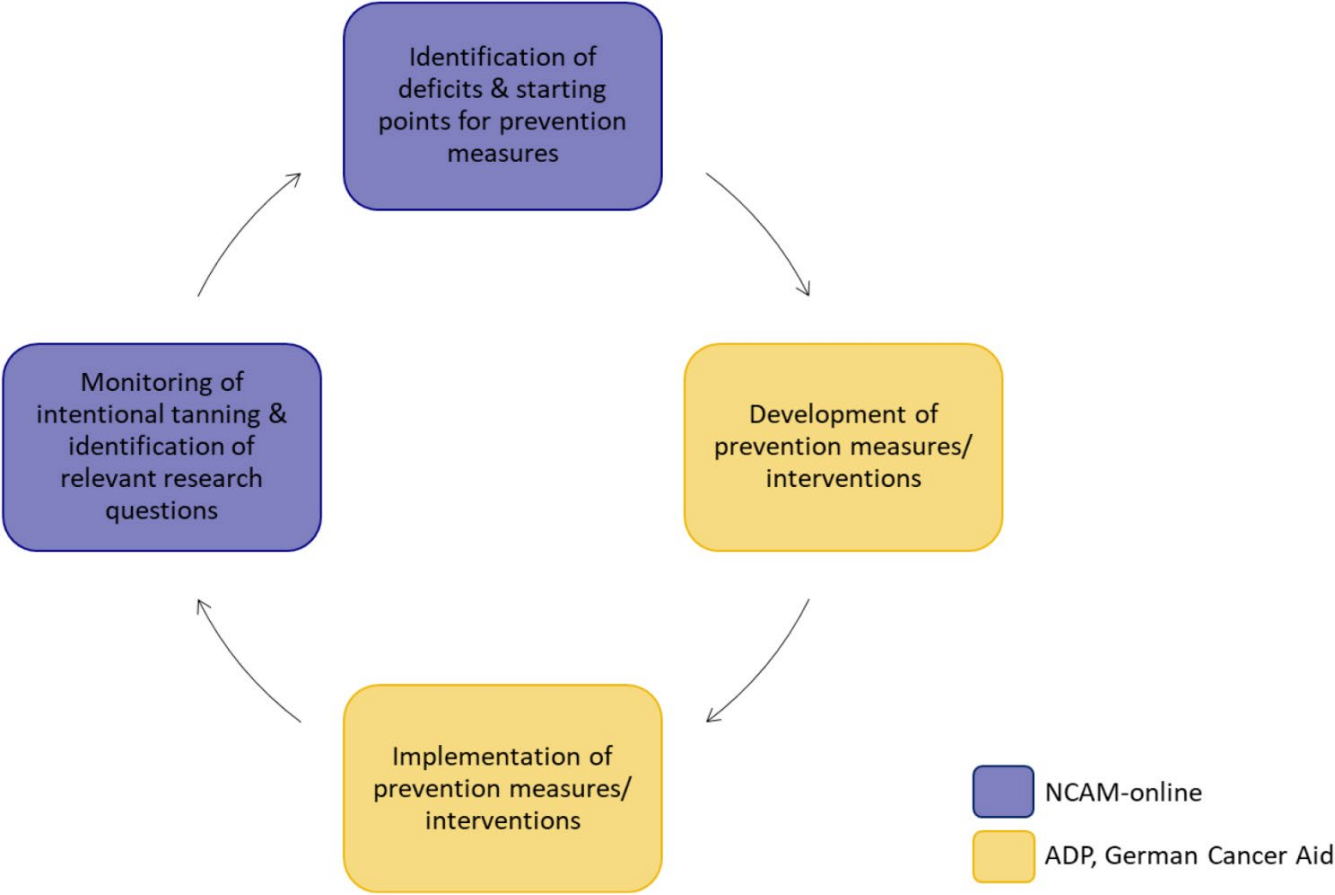
Synergistic circle of the NCAM-online. *Legend* NCAM = National Cancer Aid Monitoring; ADP = Arbeitsgemeinschaft Dermatologische Prävention (engl. : Working Group on Dermatologic Prevention)

The cooperation between NCAM-online and German Cancer Aid and ADP will be a synergistic cycle (Fig. 2): NCAM-online explores relevant questions regarding skin cancer prevention. These findings highlight potential starting points for prevention, which, in turn, may serve to develop targeted and group-specific prevention projects and campaigns. The subsequent survey waves offer an opportunity to monitor UV-related prevention and risk behaviors and provide early indications of successful prevention efforts.

The impact of the NCAM in the past will be described using three examples. First, the findings on tanning bed use led to the development of targeted prevention campaigns on social media, which were conducted by German Cancer Aid and the ADP. In addition, the “National Intervention Program on Tanning Beds” (in German: Nationales Interventionsprogramm Solarien) was developed by German Cancer Aid and ADP with the aim of banning tanning beds for cosmetic purposes in Germany. Second, the findings regarding deficits in skin cancer screening led to a revision of the mandatory training for physicians to prepare them to provide this service. The aim was to improve examination and consultation skills. All trainers in these courses were made aware of potential deficits as a part of a comprehensive recertification measure. Third, the decrease in knowledge regarding UV risks and the identified deficits in sun protection behavior were the basis for the project “Watch out at the beach”, which has been conducted by German Cancer Aid and ADP since 2022 [60]. The aim of this project is to make German beachgoers aware of the benefits of sun protection. Together with the German Lifeguard Association (Deutsche Lebens-Rettungs-Gesellschaft, DLRG), a warning system based on the UV index was implemented on certain beaches in Germany. Since the NCAM data showed that the knowledge of the UV index was low, although it is a helpful measure for adequate sun protection, the aim of the project was also to increase the knowledge and use of the UV index to help beachgoers choose appropriate sun protection.

The NCAM-online will also inform German Cancer Aid and ADP regarding findings with the aim of directly implementing these findings in skin cancer prevention and education.

## Methods/design

### Design and setting

The NCAM-online is a trend study with annual survey waves that includes a fresh sample of participants from Germany for each wave. As described above, the NCAM-online includes a core questionnaire on indoor and outdoor tanning and annually specific topics. The survey will be carried out online. An external market and social research institute will be responsible for recruiting participants following a quota plan to ensure representative data regarding sex, age, school education, and federal state. All participants provide informed consent before participation.

### Sample and sample size calculation

Between 2024 and 2028, 4,000 individuals aged 16 to 65 years will be surveyed each year. In addition to age, sufficient knowledge of German is an inclusion criterion for participation. The sample size calculation was based on the first waves of the NCAM and was already the basis for waves 2019 to 2022. The sample size was optimized according to the German Guidelines for Good Epidemiologic Practice for the primary research question, which is current tanning bed use. For the calculation, the procedure PROC POWER in the statistics software SAS was used (specification: ONESAMPLEFREQ TEST, alpha = 0.05, power = 80%). The calculation was conducted by a statistician from the Medical Faculty Mannheim, Heidelberg University.

### Development of questionnaires

The items of the core questionnaire were already used in the eight previous NCAM waves [21, 24–26]. The questionnaire was validated in the first nationwide survey (SUN-Study 2012), which included extensive pretests (cognitive interviews to analyze content validity and a test-retest survey to investigate reliability). The core questionnaire includes assessments of tanning bed use (ever use, current use, place of last use), reasons for tanning bed use, individual skin cancer risk (skin type, frequency of sunburn before the age of 15, number of nevi (> 40), malignant melanoma, malignant melanoma in family), risk perception of UV exposure, intentional tanning in the sun (on a weekday, on the weekend, on holidays), and sociodemographic characteristics.

In addition to the core questionnaire, annual specific focus themes will be surveyed as described above (wave 9: use of sun protection measures and personality structures; wave 10: climate change and active traveling outdoors; wave 11: sun protection in children; and wave 12: skin cancer screening and self-examination of the skin). The specific survey items will be developed during the project and will be approved by the ethics committee. Whenever possible, validated questionnaires will be used. In addition, all questions will be examined in a comprehensive cognitive pretest (*n* = 15 interviews before each wave). In this pretest, the understandability of questions will be checked (comprehension probing), the reliability of the answers will be examined (confidence rating), and the answer categories will be optimized (category selection probing). Based on the results of the cognitive interviews, the questionnaires may be revised and subjected to an additional pretest before use. The completion of the online survey will take approximately 15 min.

### Statistical analysis

The raw data will be examined for plausibility and representativeness. Then, the data will be edited and prepared for analysis by (re)coding variables. The data will be prepared and analyzed using IBM SPSS Statistics (version 29).

In the first step, frequencies, bivariate associations, and differences will be analyzed. Different tests will be used based on the structure of the variables (chi² tests for nominal and ordinal data; t tests and ANOVAs for group differences of parametric variables; Mann‒Whitney U tests and Kruskal‒Wallis H tests for group differences of nonparametric variables). Based on these findings, linear and logistic regressions will be performed. In addition, cluster and factor analyses can be used to reduce complexity. In addition to wave-specific analyses, trends in the prevalence of current tanning bed use will be analyzed. For all analyses, a p value < 0.05 was considered to indicate statistical significance.

## Discussion

The NCAM-online plays an important role in skin cancer prevention in Germany. As in the previous eight waves, the NCAM-online will provide current data on UV-related risk behaviors, sun protection behaviors, and related aspects to help identify target groups and important issues for future prevention. This will not only help develop targeted education but will also continue to provide information for structural prevention (e.g., legislation on indoor tanning).

This study has several limitations. First, we collect annual cross-sectional data. These data do not allow us to draw causal conclusions. However, the instrument is able to provide trend data on UV-related risk behavior and allows the exploration of additional topics that can be relevant for skin cancer prevention. Second, our sample is selective, but quota sampling is used to be as representative as possible for Germany regarding sex, age, school education, and federal state. Third, as in other population-based survey studies, we cannot exclude social desirability and recall biases. However, previous test-retest analyses have shown good reliability for questions on tanning bed use [18], and we will mainly use instruments that have been validated in previous studies. In addition, all new questions will be examined in cognitive pretests before each survey wave.

Overall, the NCAM-online is based on comprehensive prior work and experience. In particular, cooperation with key players in the prevention of skin cancer is a strength of the NCAM-online. This allows the direct transfer of scientific findings to prevention practices.

## Data Availability

The datasets used and/or analyzed during the current study are available from the corresponding author upon reasonable request.

## Abbreviations

ADP: Arbeitsgemeinschaft Dermatologische Prävention
DLRG: Deutsche Lebens-Rettungs-Gesellschaft
NCAM: National Cancer Aid Monitoring
NCAM-online: National Cancer Aid Monitoring online
UV: Ultraviolet
